# Unraveling the Interplay Between Alcohol, Immunity, and Gastric Cancer: A Genomic Approach

**DOI:** 10.1002/fsn3.71260

**Published:** 2025-11-24

**Authors:** Ke‐Jie He, Haitao Wang

**Affiliations:** ^1^ The Quzhou Affiliated Hospital of Wenzhou Medical University, Quzhou People's Hospital Quzhou Zhejiang China; ^2^ The School of Clinical Medical Sciences Southwest Medical University Luzhou Sichuan China

**Keywords:** alcohol intake, gastric cancer, immune cell profiles, Mendelian randomization, molecular classification

## Abstract

Gastric cancer poses a major global health burden. Immune evasion is a hallmark of gastric tumorigenesis, yet determinants of anti‐tumor immunity and its interactions with cancer development remain unclear. Dietary patterns strongly influence gastric cancer risk, but studies typically examine broad lifestyle factors rather than immune cell populations. We employed Mendelian randomization to investigate potential causal effects of alcohol intake and over 700 immune cell profiles on gastric cancer susceptibility using summary statistics from genome‐wide association studies. Multivariable Mendelian randomization and mediation analyses assessed combined effects. SNP annotation characterized immunophenotype‐associated genes. Integrative profiling classified gastric cancer subgroups. Mendelian randomization identified immune signatures associated with altered gastric cancer risk, including increased risk from alcohol intake, CD28 on resting Treg and CD62L‐ HLA DR++ monocyte AC. Multivariable Mendelian randomization provided evidence that higher natural killer cell percentages may lower cancer risk, while naive‐mature B cells potentially mediated alcohol's effect. Annotation implicated 104 risk‐linked genes in tumorigenesis. Consensus clustering stratified patients by survival dependent on coordinated immunophenotypic programs. Focusing on ACOXL, elevated tumor expression was associated with poorer prognosis. Our study provides genetic and functional evidence linking environmental exposures, immune cell compositions and gastric cancer risk. Findings advance comprehension of disease etiology and establish an integrated immunogenomic framework with implications for precision immunotherapies and disease prevention strategies.

## Graphical abstract

This study investigates the genetic and immune factors contributing to gastric cancer risk, with a focus on alcohol consumption and immune cell profiles. Mendelian randomization identified immune signatures associated with increased cancer risk, particularly through alcohol intake and specific immune cell alterations. The study also highlighted the role of natural killer cells in lowering cancer risk and the potential mediating effect of B cells, providing insights for targeted therapies.

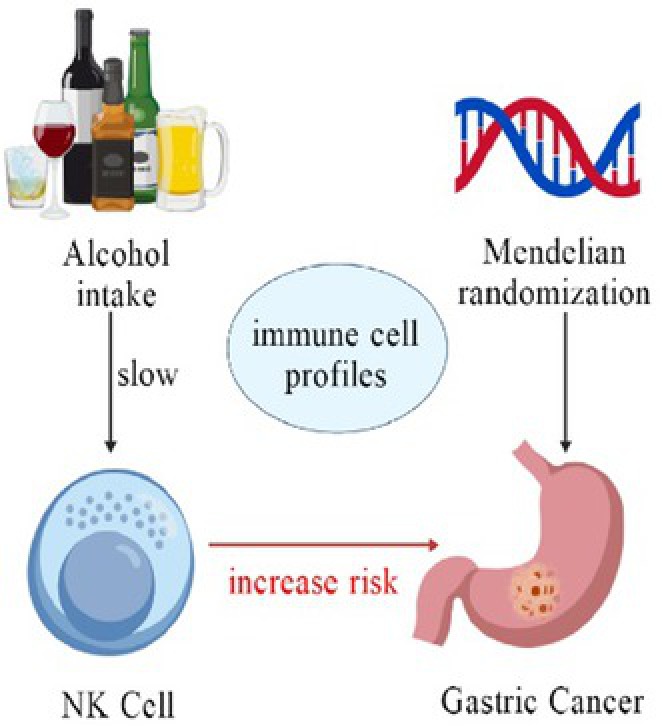

## 1 Introduction

Gastric cancer poses a substantial global health burden and remains a leading cause of cancer mortality worldwide (Rahman et al. 2014; Van Cutsem et al. 2016). Despite advances in treatment, prognosis remains poor due in part to late‐stage diagnoses and resistance to conventional therapies (Al‐Batran et al. 2017; Califano and Alvarez 2017; Thompson et al. 2017). Immune evasion represents a hallmark of gastric tumorigenesis, yet precise determinants of anti‐tumor immunity and interactions between the immune system and cancer development remain incompletely understood (Hanahan and Weinberg 2011; Schreiber et al. 2011).

Epidemiological studies have shown dietary patterns strongly influence gastric cancer risk (Chyou et al. 1990; Ko et al. 2013), with Western diets high in processed and red meats and alcohol drinking linked to increased susceptibility (Tramacere et al. 2012; Zhu et al. 2013). However, it is uncommon to find studies that have specifically investigated the effects of dietary exposures on gastric cancer etiology by stratifying the effects across distinct immune cell populations. The consumption of alcohol induces an inflammatory response and leads to a state of hyper‐inflammation, which is responsible for its detrimental effects (Heo et al. 2019; Nagy et al. 2016; Saha et al. 2015; Verma et al. 2016). Some studies demonstrated the impairment of Th1‐mediated delayed hypersensitivity (DTH) reactions by alcohol in a murine model that mimics human alcoholism, highlighting the involvement of different immune cells and their interactions in the effects of alcohol on the immune system (Latif et al. 2002; Lundberg and Passik 1997). Moreover, the consumption of alcohol leads to the suppression of natural killer (NK) cell cytolytic activity (Mathews et al. 2016; Meadows et al. 1992; Nagy et al. 2016; Spitzer and Meadows 1999). A precise delineation of the immune landscape in gastric cancer and its modulation by modifiable environmental determinants could offer novel opportunities for tumor immunoprevention and immunotherapy.

Here we employ a systems‐level approach leveraging large‐scale genomic and epidemiological datasets to comprehensively map associations between immune cell compositions, alcohol intake, and gastric cancer risk. By applying Mendelian randomization, a technique using genetic variation as instrumental variables to infer causation, we identify specific immune signatures differentially tied to gastric cancer susceptibility at single‐cell resolution. Follow‐up annotation elucidates host genes underlying implicated loci for insights into biological mechanisms.

Moreover, through integrative multi‐omic profiling we develop an immune‐informed molecular taxonomy of gastric cancer stratifying patient survival. Focusing on ACOXL, we characterize its clinical importance and functions. Collectively, our findings offer new perspectives on environmental influences on immunity and gastric tumorigenesis with implications for customized screening, prevention and treatment approaches.

## 2 Materials and Methods

### 2.1 Study Design

Our research aimed to uncover causal links between lifestyle habits, biological makeup, and stomach cancer risk. Using a technique called Mendelian randomization (MR), we analyzed the potential impacts of alcohol intake and over 700 immune cell profiles on gastric cancer susceptibility.

MR leverages natural genetic markers as proxies to infer relationships, provided its underlying assumptions hold. Namely, the genetic factors strongly influence exposures, are independent of confounding variables, and affect outcomes solely through exposures without alternate routes (Bowden et al. 2016; Burgess et al. 2017; Hartwig et al. 2017; Yavorska and Burgess 2017).

We drew on data from genome‐wide association studies approved by ethics boards with participant consent. For 731 immune signatures across seven categories, we identified reliable, standalone genetic instruments from previous broad genomic scans. We then tested the signatures' ties to stomach cancer using an independent data set to avoid bias. Given the strong correlation structure among many of these immunophenotypes (e.g., markers within the same cellular compartments or panels), conventional multiple‐testing procedures that assume independence, such as Bonferroni correction, are likely to be overly conservative and inflate type II error in a discovery‐oriented MR screen. Accordingly, we prospectively designated this phenome‐wide screen as hypothesis‐generating and report nominal two‐sided P values together with effect sizes and 95% confidence intervals, prioritizing biological coherence and methodological robustness over strict family‐wise error control.

To ensure sufficient statistical power for the Mendelian randomization analyses, we performed power calculations based on the expected effect sizes and the sample size of each GWAS dataset. These calculations were used to determine the minimum detectable effect size with a given level of statistical significance and power (80%) for each of the immune traits and gastric cancer associations. This approach allowed us to assess whether the study was adequately powered to detect meaningful causal relationships.

Our re‐analyses targeted alcohol consumption and the 731 cell profiles parsed from subsets related to innate and adaptive immunity. By carefully employing MR and validating our proxies' strength and singularity, we sought to clarify plausible causal links between these exposures, immunity constituents, and gastric cancer risk. Our findings offer new genetic evidence for how certain immune components could shape stomach cancer development, with implications for custom treatment and prevention strategies. Overall, interrogating exposures and biology across multiple levels through MR enhanced recognition of modifiable disease determinants.

### 2.2 Data Sources

Genome‐wide association data for stomach cancer (GC) risk were drawn from a prior examination of over 476,116 individuals with European heritage led by Sakaue et al. (Sakaue et al. 2021). We accessed publicly available summary‐level genetic data from the MRC Integrated Epidemiology Unit (MRC‐IEU) Open GWAS database (https://gwas.mrcieu.ac.uk/), which provides open‐access genetic association data without requiring specific permissions. Statistics on stomach cancer (GC) were retrieved from dataset ieu‐b‐42 (*n* = 77,096). Statistics on alcohol consumption were retrieved from dataset ieu‐b‐4834 (*n* = 83,626). Publicly accessible GWAS summary results for the 731 immunophenotypes were culled from the GWAS Catalog (accession numbers GCST0001391 through GCST0002121), divided into absolute cell counts (118 traits), fluorescence intensity indicating surface antigen levels (389 traits), morphological parameters (32 traits), and relative cell amounts (192 traits). Namely, the fluorescence intensity, absolute count and relative count libraries characterized B cell, regular dendritic cell, mature T cell subsets, monocyte, myeloid cell, TBNK (T cell, B cell, innate killer cell) and regulatory T cell characteristics, while morphological parameters covered dendritic and TBNK cell metrics.

The original immunophenotype GWAS leveraged genotype information from around 22 million SNPs on high‐density arrays in 3757 Europeans without cohort overlap (Orrù et al. 2020; Sidore et al. 2015). Imputation was conducted using a Sardinian reference panel followed by connection screening adjusting for sex, age and age‐squared as cofactors. This ensured no confounding from hidden population structure and afforded robust instrumental factors for our causal deduction analyses of GC susceptibility.

### 2.3 Multivariable MR Methods (MVMR) and Decomposing Mediated Effects

For our multivariable Mendelian randomization (MVMR), we employed the inverse‐variance methodology as our primary method (Sanderson 2021; Zheng et al. 2017). In the initial analysis, we presumed zero cross‐correlation between SNP relationships. We tested this presumption using a range of covariance values.

The overall impact of an exposure on an outcome can be divided into indirect (i.e., mediated via a causal mediator) and direct (i.e., not through the mediator) parts. To decompose the total effect of alcohol consumption on stomach cancer risk, we calculated the indirect effect through each mediator singly using the product technique. The proportion mediated was quantified by dividing the indirect effect by the total effect. Confidence intervals were estimated utilizing the delta process (Sanderson 2021).

### 2.4 Selection of Instrumental Variables (IVs)

For the immune traits, we set a genome‐wide significance threshold of *p* < 1e‐5, which is a commonly used threshold in previous research focusing on immune‐related SNPs. This threshold was chosen to balance sensitivity and specificity in detecting associations that are likely to be biologically meaningful without overwhelming the analysis with false positives. This is a standard threshold adopted in the literature for immune‐related traits, which tend to exhibit more complex genetic architectures compared to disease traits. In contrast, for gastric cancer (GC), we applied a more stringent threshold of *p* < 5e‐8, which is consistent with the widely accepted threshold for GWAS focused on disease outcomes. This stricter threshold helps reduce the risk of including spurious associations, as GC is a complex disease with a higher demand for robustness in SNP‐outcome connections.

To further mitigate the potential for weak instrument variable bias, we carefully selected SNPs by considering both their genome‐wide significance and their linkage disequilibrium (LD) structure. We used PLINK software (v1.90) to prune out linked single nucleotide polymorphisms (SNPs) and identify independent ones with an LD *r* ^2^ below 0.1 within 500 kb. LD *r* ^2^ values came from the 1000 Genomes Project panel (Orrù et al. 2020).

For gastric cancer, we adhered to the *p* < 5e‐8 cutoff to ensure robust SNP‐outcome associations, and we selected independent GC risk‐associated SNPs with an r^2^ under 0.005. Once we finalized the instrumental variants, we harmonized the genome‐wide association study (GWAS) data by extracting the GC GWAS data corresponding to each variant we retained. This process enabled us to examine the specific contribution of GC risk driven by the genetic instruments we identified for immune profiles, ensuring that the assumptions for two‐sample Mendelian randomization were met.

Overall, through a careful selection of SNPs and alignment of the GWAS data, our study design allowed for a thorough investigation of potential causative immune cell signatures for GC risk, while adhering to best practices for inferring genotype–phenotype relationships.

### 2.5 SNP Annotation

We used the online tool g:SNPense to annotate genetic variants. This resource maps human SNPs listed by rs IDs to gene names through integrated analyses of chromosomal positions and predicted functional effects from Ensembl Variation. Specifically, g:SNPense pulls out relevant gene contexts for input variants that intersect protein‐coding gene loci as defined by the Ensembl gene set. Leveraging g:SNPense made it easier to characterize potential functional roles and biological pathways for variants linked to immune cell traits and gastric cancer risk. These annotations gave us molecular insights into how certain immune cell populations could influence gastric tumor susceptibility as suggested by our Mendelian randomization analyses. Overall, g:SNPense was really helpful—it enhanced how we interpreted the genetic findings by mapping variants to their matching gene targets encoded in the human reference genome. This resource made characterizing variant functions and biological mechanisms more straightforward, providing details that supplemented the statistical inferences from our epidemiology study.

### 2.6 Construct Prognostic Signature of Immunophenotypes

We developed a prognosis model for gastric cancer (GC) based on immune gene expression signatures. The model was constructed using RNA sequencing data and survival outcomes from 375 GC patients in The Cancer Genome Atlas (TCGA).

After preprocessing the transcript per million (TPM) values via log2 transformation with a pseudocount of 1 to reduce positive skewness, we performed least absolute shrinkage and selection operator (LASSO) regularized Cox regression with 10‐fold cross‐validation to identify a parsimonious set of immune genes predictive of patient survival. This analysis was done using the R package glmnet.

The prognostic value of the identified immune signature was evaluated via Kaplan–Meier analysis and log‐rank test. Kaplan–Meier curves with 95% confidence intervals were generated using the R survival package to stratify patients into high‐ and low‐risk groups based on a median split of signature scores. Direct comparison of survival outcomes between the two risk groups determined the ability of the signature to discriminate prognosis.

Statistical significance was set at *p* < 0.05. In summary, our computational workflow leveraged both the transcriptomic and clinical outcome data from TCGA patients to develop an immunogenomic classifier for predicting prognosis in GC.

### 2.7 Identification of Potential Subtypes

We conducted unsupervised clustering to reveal novel molecular subgroups in gastric cancer (GC). Transcriptomic profiles from The Cancer Genome Atlas were analyzed. Samples were randomly divided into a larger discovery set (80%) and a smaller validation set (20%). ConsensusClusterPlus iteratively clustered the discovery set 100 times. Stable clusters emerged, highlighting key expression patterns. Heatmaps visualized differentially expressed genes across clusters. When over 1000 genes met significance, the most variably expressed top 25% were retained.

The validated set then determined if these discovery clusters accurately predicted new cases' molecular profiles. Repeated sampling reinforced cluster robustness/reproducibility. External data further corroborated biological relevance, as clusters stratified GC into distinct pathologies. RNA sequencing thus uncovered GC's intrinsic heterogeneity through an unbiased classification approach.

Validation on independent patients supported clinically meaningful subtypes. This multidimensional transcriptomic analysis differentiated GC at the molecular level, deepening our understanding of disease pathogenesis for improved precision oncology.

### 2.8 Cell Culture and Transfection

We conducted experiments using gastric cancer cells to examine the role of the ACOXL gene. The MGC‐803 line was obtained from a trusted source and shown to be clear of contamination.

Standard cell culture methods were employed for stable expansion. Cells thrived in nutritive media under physiological temperatures, allowing controlled proliferation.

Gene expression was modulated using RNA interference technology. MGC‐803 cells were transfected with either ACOXL‐targeting siRNA or a negative control. A lipid‐based agent facilitated genetic alteration per manufacturer guidelines.

Analyses after 24 h revealed the functional effects of ACOXL suppression. This innovative experimental design established a cellular system to explore oncogenic impacts.

By selectively depleting ACOXL, we gained new mechanistic insights with therapeutic relevance. The results further our understanding of gastric tumorigenesis and identify potential clinical applications warranting preclinical development. In summary, this study provides a platform to examine ACOXL biology and uncover novel treatment avenues for gastric cancer.

### 2.9 Cell Apoptosis

We conducted functional studies to gain insight into ACOXL's influence on gastric cancer cell behavior and fate. Transfected cells harvested after 2 days were labeled using a commercial kit and analyzed by flow cytometry to quantify the apoptotic fraction displaying marker signals. All experiments were performed in triplicate for accuracy.

## 3 Results

### 3.1 Genetic Evidence for a Causal Link Between Alcohol Intake and Increased Risk of Gastric Cancer

We used a Mendelian randomization approach to investigate the causal effect of alcohol intake on gastric cancer risk. Specifically, we applied the inverse variance weighted (IVW) method to summarize data from multiple genetic variants associated with alcohol consumption.

**FIGURE 1:**
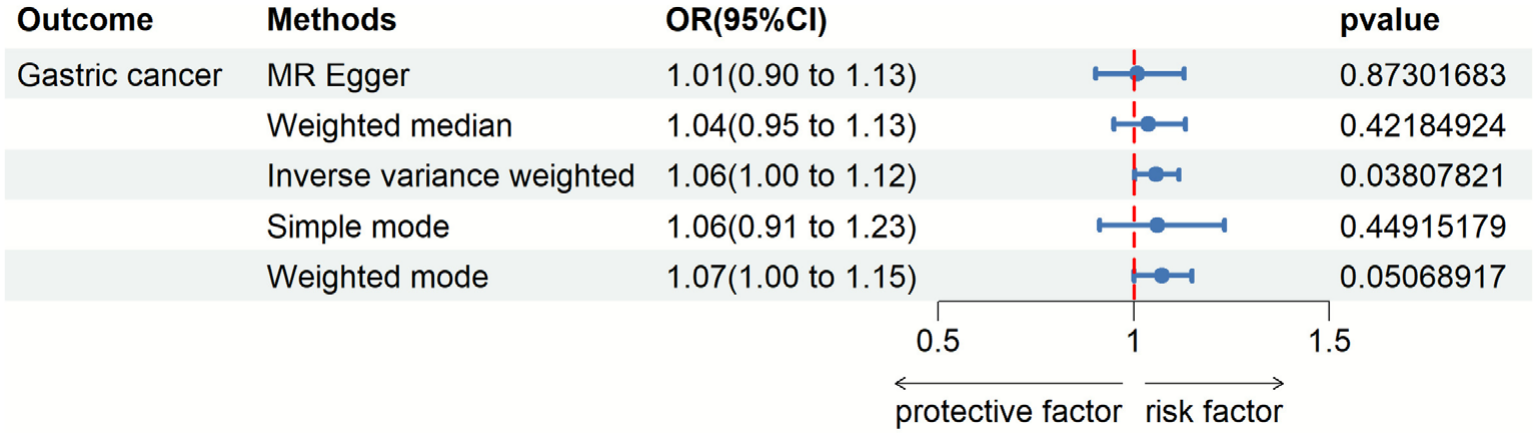
Causal effect of alcohol intake on gastric cancer risk. Using Mendelian randomization and inverse variance weighted method, the analysis found a positive relationship between alcohol intake and gastric cancer odds. Results showed each additional alcohol unit increased cancer risk 6%, supporting a causal role.

The IVW analysis found a statistically significant positive relationship between genetically instrumented alcohol intake and odds of gastric cancer (Figure 1). Results indicated that for each additional unit of alcohol consumed, the odds of gastric cancer increased by 6% (odds ratio 1.06, *p* = 0.038). Confidence intervals (1.003 to 1.115) did not cross the null value of 1, providing evidence for a causal risk‐elevating effect rather than no effect or protection.

As the most commonly used Mendelian randomization method, IVW directly estimates the causal effect size assuming instrument validity. Taking the results at face value, the genetic data suggest higher alcohol intake may causally increase gastric cancer risk by around 6%.

In conclusion, the findings of this instrumental variable analysis provide novel evidence that alcohol consumption plays a contributory role in gastric carcinogenesis. The specific information is presented in Supporting Information S1. Overall, the study sheds new light on an important environmental risk factor for gastric cancer.

### 3.2 Identifying Key Immunophenotypes Linked to Gastric Cancer Risk Through Mendelian Randomization Analysis

**FIGURE 2:**
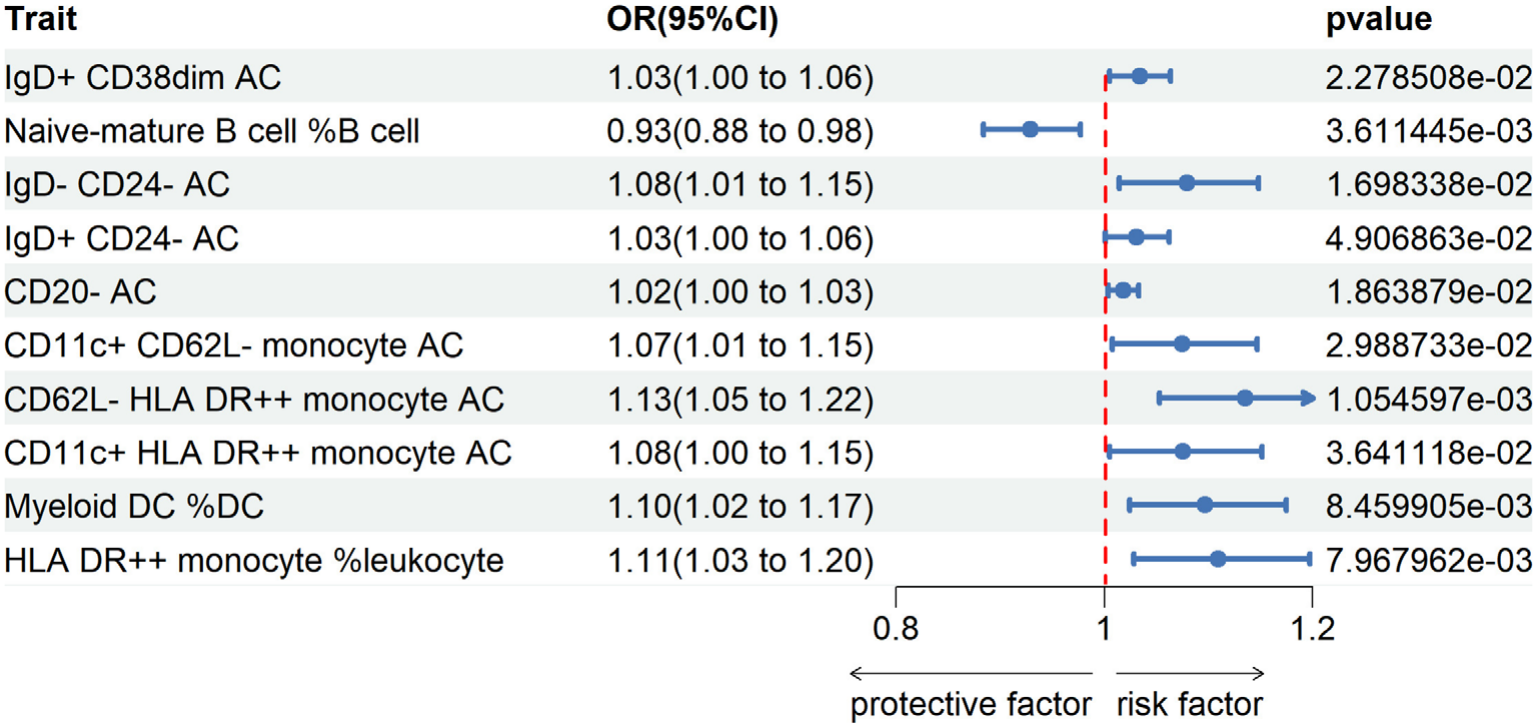
Causal links between immunophenotypes and gastric cancer risk. Mendelian randomization using inverse variance weighting identified 19 protective and 22 risk‐associated immune profiles. Notably, top risk phenotypes included alterations in DN (CD4‐CD8‐) AC, CD62L‐ HLA DR++ monocytes, CD28 on Tregs, HLA DR++ monocyte percentage, and myeloid DC percentage. Conversely, top protective phenotypes featured different cell markers. Rigorous analysis identified specific immune cell types modulating GC risk and potential immunotherapies.

We conducted a two‐sample Mendelian randomization study to explore causal links between immunophenotypes and gastric cancer (GC) risk. Inverse variance weighting identified 19 protective and 22 risk‐associated profiles (Figure 2). Because this screen encompassed 731 correlated traits, results are reported with nominal P values and interpreted as hypothesis‐generating rather than confirmatory.

Notably, the top five risk phenotypes were: (1) DN (CD4−CD8−) AC (TBNK panel; odds ratio [OR] 1.14, 95% confidence interval [CI] 1.06–1.24, *p* = 0.0006), (2) CD62L‐ HLA DR++ monocyte AC (cDC panel; OR 1.13, CI 1.05–1.22, *p* = 0.001), (3) CD28 on resting Treg (Treg panel; OR 1.12, CI 1.02–1.23, *p* = 0.01), (4) HLA DR++ monocyte %leukocyte (TBNK panel; OR 1.10, CI 1.02–1.19, *p* = 0.007), and (5) Myeloid DC %DC (cDC panel; OR 1.09, CI 1.02–1.17, *p* = 0.008). Further details are provided in Supporting Information S2.

Conversely, the top five protective immunophenotypes were characterized by: (1) CD8 on TD CD8br cells (Maturation stages of T cell panel; OR 0.91, 95% CI 0.85–0.98, *p* = 0.03), (2) CD20 on IgD− CD38− (B cell panel; OR 0.91, 95% CI 0.84–0.99, *p* = 0.04), (3) CD20 on unsw mem (B cell panel; OR 0.91, 95% CI 0.85–0.97, *p* = 0.03), (4) Naive CD4+ AC (Maturation stages of T cell; OR 0.90, 95% CI 0.83–0.96, *p* = 0.03), and (5) IgD on IgD+ (B cell panel; OR 0.88, 95% CI 0.82–0.95, *p* = 0.03) as estimated by IVW.

Rigorous analytical techniques highlighted particular immune cell types that modulate GC risk. Insights into immune cell behaviors offer new avenues for prevention and treatment of GC.

Overall, this instrumental variable analysis uncovered novel relationships between immune signatures and GC susceptibility. The findings provide mechanistic understanding and highlight potential immunotherapeutic strategies warranting further preclinical investigation.

### 3.3 Assessment of Potential Causal Effects of Gastric Cancer Onset on the Immune System Using Two‐Sample Mendelian Randomization

**FIGURE 3:**
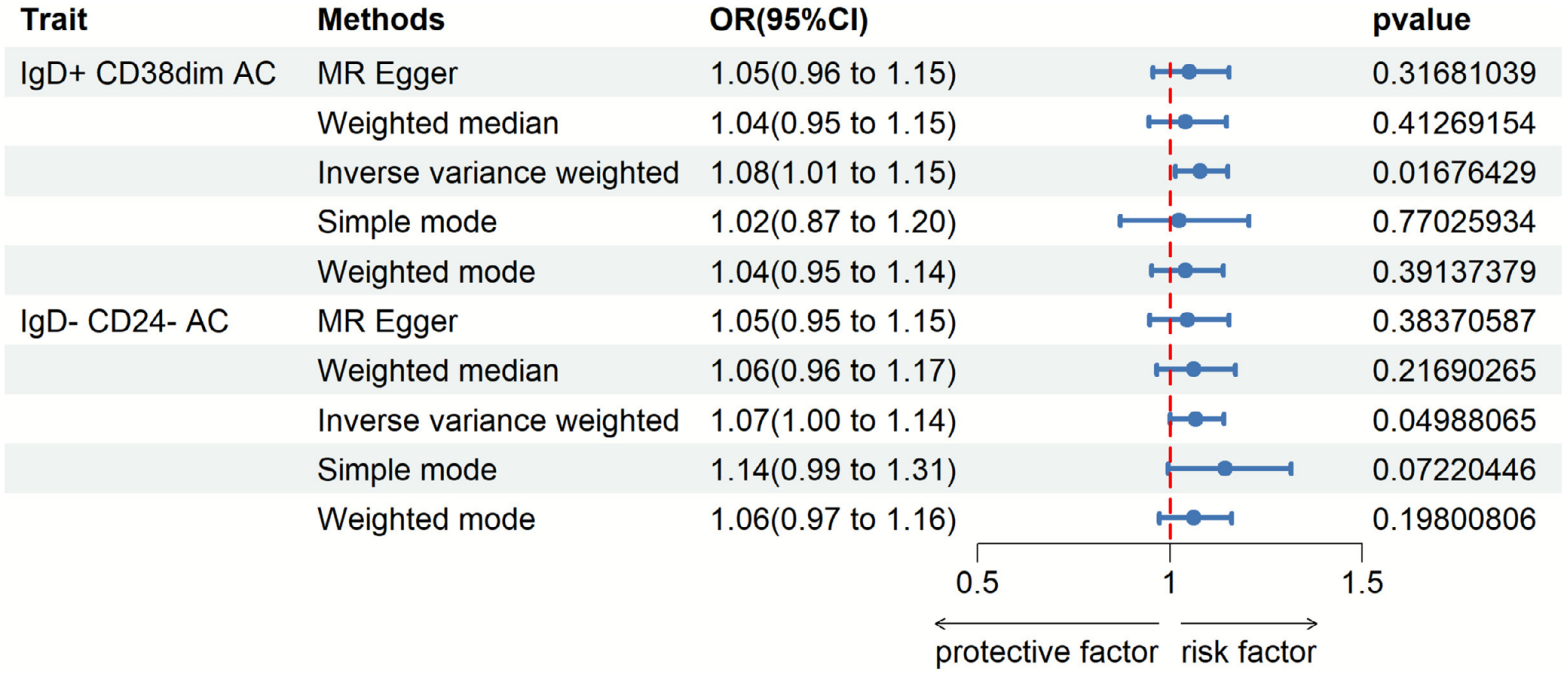
Effects of gastric cancer onset on the immune system. Two‐sample Mendelian randomization identified initial associations between GC and increased IgD+CD38dim activated B cells and IgD‐CD24‐ activated B cells. However, more robust MR methods did not support causal links. Rigorous evaluation found no confirmation of causal immune modulation by GC based on current evidence. Larger datasets are needed to clarify relationships.

We employed two‐sample Mendelian randomization (MR) to investigate potential causal impacts of gastric cancer (GC) onset on the immune system. Initial inverse variance weighting identified associations between GC and increased IgD+CD38dim activated B cells as well as IgD−CD24− activated B cells (Figure 3). Further details are available in Supporting Information S3.

However, the MR‐Egger and weighted median methods did not support causal links. While preliminary analyses suggested immune alterations relating to GC, more robust methodologies highlighted potential biases not accounted for.

Rigorous evaluation prevents confirmation of causal immunophenotype modulations downstream of gastric tumorigenesis based on current evidence. Larger datasets may clarify relationships between malignancy and immune microenvironment in the future.

In summary, this instrumental variable analysis employing multiple analytical techniques was unable to substantiate that GC causally influences the immune system. Continued research employing improved methods and sample sizes remains warranted.

### 3.4 Examining Potential Causal Effects of Alcohol Intake on Immune Cell Phenotypes

We employed Mendelian randomization to investigate potential causal effects of alcohol intake on immune cell phenotypes.

Inverse variance weighting detected a statistically significant negative association between genetically predicted alcohol levels and the proportion of naive‐mature B cells. Results indicated that for each alcohol unit increase, the naive‐mature B cell fraction decreased 7.4% (OR 0.9258, *p* = 0.0187). The non‐overlapping 95% CI (0.8682 to 0.9873) provided evidence of a causal effect.

A marginally significant negative relationship was also found for natural killer (NK) cells, with alcohol lowering their frequency by 6.2% (OR 0.9377, *p* = 0.0359). Again, the 95% CI (0.8831% to 0.9957%) did not span the null value.

**FIGURE 4:**
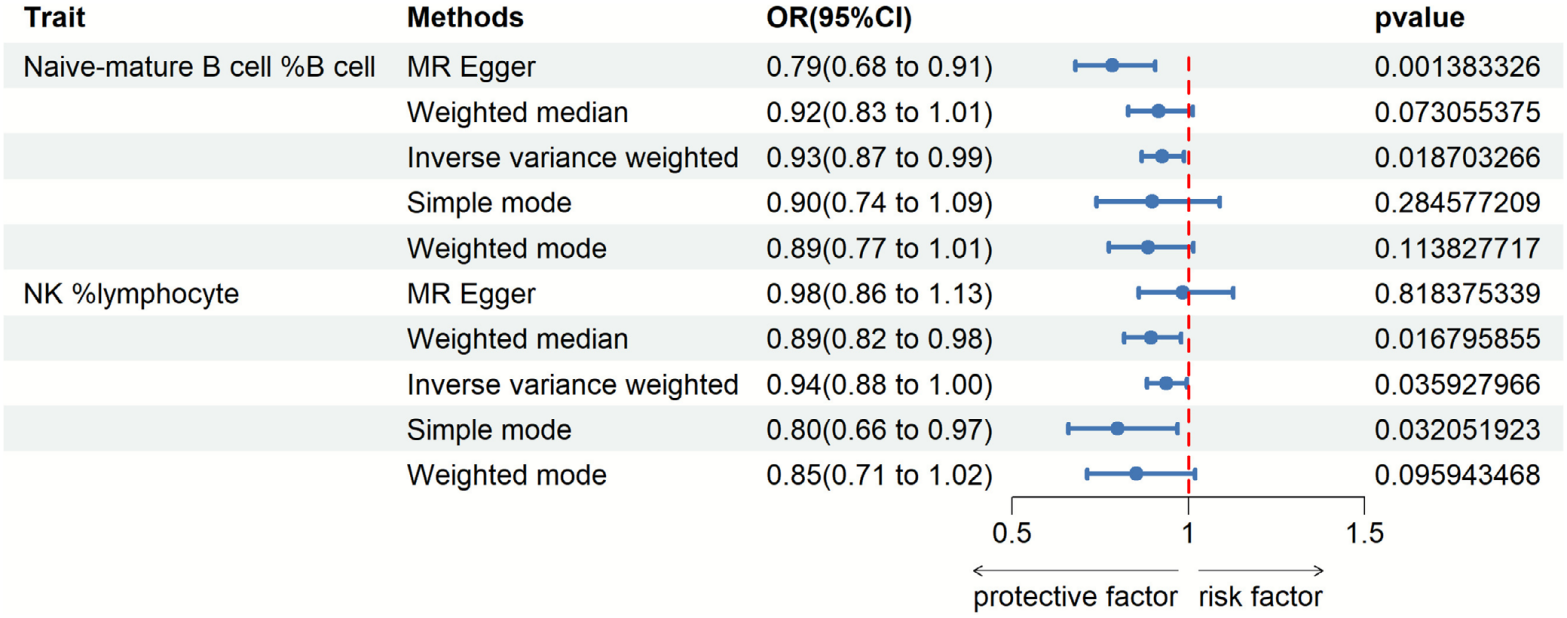
Effects of alcohol intake on immune cell phenotypes. Mendelian randomization using inverse variance weighting detected a significant negative association between alcohol levels and the proportion of naive‐mature B cells, with a 7.4% decrease per alcohol unit. A marginally significant negative association was also found for NK cell frequency. Genetic data tentatively suggest causal reductions in specific immune cell populations with alcohol intake, warranting validation.

Adopting the more reliable IVW estimates as the primary analysis, the genetic data tentatively suggest alcohol intake could directly reduce both the percentage of naive‐mature B cells and NK cells in a causal manner (Figure 4, Supporting Information S4). Replication in larger studies is needed to validate these exploratory findings.

In summary, using a Mendelian randomization approach, this study generated novel hypotheses regarding potential immunological mechanisms linking alcohol and disease risk. Further investigation is warranted.

### 3.5 Effect of Alcohol Consumption and Immune Cells on Gastric Cancer by Multivariable Mendelian Randomization (MVMR)

The results of a multivariable Mendelian randomization (MVMR) analysis examining the potential causal effects of three exposures—naive‐mature B cell percentage, natural killer cell percentage, and alcohol consumption—on the risk of gastric cancer. The MVMR approach provided genetic evidence that higher natural killer cell percentages may lower gastric cancer risk, while greater alcohol consumption may slightly increase risk based on these data. No relationship was found for naive‐mature B cells and gastric cancer (Table S1).

While naive‐mature B cell percentage appeared to have a significant causal effect when examined independently through two‐sample Mendelian randomization, the multivariable MR analysis did not detect an association. This discrepancy could potentially be explained by mediation. Specifically, it is possible that naive‐mature B cell percentage mediates the causal effect of alcohol consumption on gastric cancer risk.

When alcohol intake and B cell trait were both included in the MVMR model, their individual estimates may have been attenuated if the B cell trait functions along the causal pathway from alcohol to gastric cancer. This suggests naive‐mature B cells could play an intervening role, whereby the genetic effect of alcohol on raising B cell levels in turn influences gastric cancer risk.

The observation lays the groundwork for follow‐up mediation analyses to formally test if the association between alcohol and gastric cancer is mediated through naive–mature B cells. Mediation MR techniques could help discern whether targeting B cell populations could help mitigate cancer risk attributed to alcohol exposure.

Overall, our hypothesis provides a valuable interpretation for the divergent single and multivariable MR results. Exploring mediation more rigorously could help clarify the potentially complex interplay between alcohol consumption, immune alterations and gastric carcinogenesis. This warrants further investigation.

### 3.6 Estimate of the Effect of Alcohol Consumption on Gastric Cancer Explained by Naive‐Mature B Cells and NK Cells

We further examined the proportion of the effect of alcohol consumption on gastric cancer risk that is explained through two immune cell traits: naive‐mature B cell percentage and natural killer cell percentage (Table S2).

For naive‐mature B cell percentage: The analysis estimates that 10.97% of the total effect of alcohol on gastric cancer is mediated through this immune trait. The 95% confidence interval ranges from 3.58% to 18.36%, not crossing zero, providing evidence of a true mediating role. The *p*‐value of 0.035346 confirms this mediation effect is statistically significant.

For natural killer cell percentage: The proportion mediated was smaller at 5.10%. However, the confidence interval from 0.008235 to 0.093797 overlaps zero, indicating weaker evidence of mediation through NK cells based on the available data. The *p*‐value of 0.065856 also suggests this relationship does not meet the threshold for statistical significance.

In summary, this mediation analysis provides genetic support that approximately 11% of the causal influence of alcohol on gastric cancer risk operates through downstream effects on naive‐mature B cell proportions. Further studies are warranted to validate NK cells as a potential mediator. Overall, this analysis quantifies the extent to which the immune system may underlie associations between dietary exposures and cancer.

### 3.7 SNP Annotation

We conducted follow‐up analyses to characterize single‐nucleotide polymorphisms (SNPs) meeting genome‐wide significance for the top five risk‐elevating and protective immunophenotypes related to gastric cancer (GC).

Annotation of loci implicated 104 host genes potentially involved in GC pathology. Further details are available in Supporting Information S5.

Beyond identifying causal immunological traits, genetically inferring putative genes represents an important step toward elucidating biological mechanisms linking immune profiles to GC susceptibility. The findings warrant exploration of molecular pathways connecting susceptibility loci to immunophenotype regulation and disease outcome.

Elucidating disease‐relevant genetics offers targets for future experimental validation and therapeutic intervention. Continued molecular characterization utilizing well‐powered genetic datasets promises new insight into the immunogenetics of gastric tumorigenesis.

### 3.8 Classifying Gastric Cancer Through an Immune‐Informed Molecular Taxonomy

**FIGURE 5:**
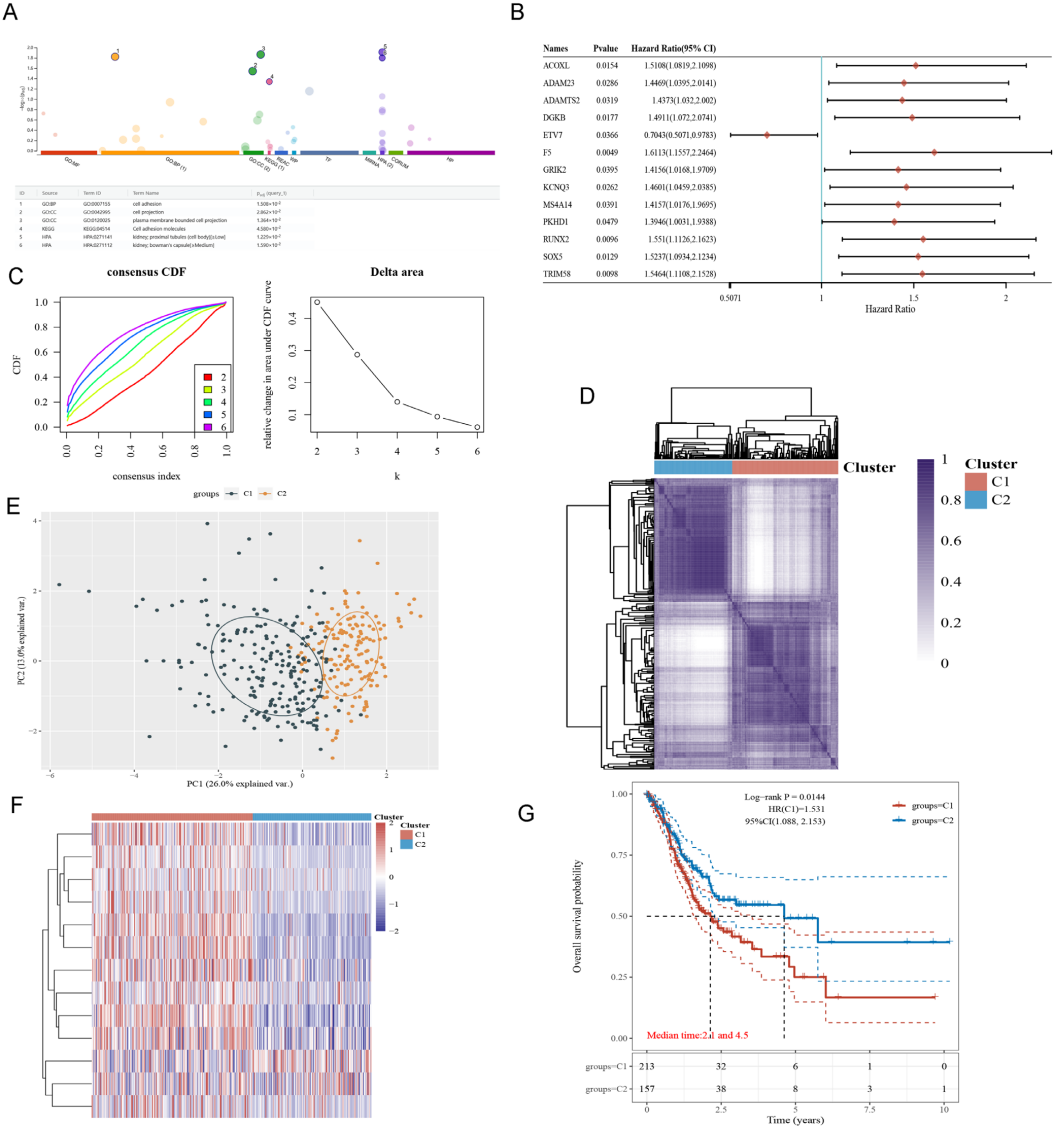
Development of an immune‐informed molecular taxonomy for gastric cancer. (A) Pathway enrichment of 104 genes linked to immunophenotypes implicated cellular communication pathways. (B) 13 prognostic genes selected based on the 104 genes. (C) Unsupervised machine learning using consensus clustering. (D) Unsupervised consensus clustering of TCGA gastric cancer RNA‐seq data stratified patients into two subgroups, C1 and C2, based on the expression of 13 prognostic genes selected by LASSO regression. (E) Principal component analysis of transcriptomic data between clusters. (F) Consensus clustering heatmap showed discrete molecular profiles between clusters. (G) Kaplan–Meier plots revealed significantly different patient prognoses associated with each cluster.

This study developed an immune‐informed classification of gastric cancer integrating multi‐dimensional omics data. Pathway enrichment of 104 genes linked to immunophenotypes (identified in previous analysis) implicated cellular communication pathways, including cell adhesion, cell projection, plasma membrane‐bounded cell projection, cell adhesion molecules (Figure 5A).

Unsupervised machine learning using consensus clustering (Figure 5C) stratified TCGA patient samples into two subgroups (C1 and C2) based on the expression of 13 prognostic genes selected through profiling (Figure 5, Supporting Information S6).

Heat mapping (Figure 5F) and principal component analysis (Figure 5E) showed discrete molecular profiles between clusters. Validation with Kaplan–Meier plots and log‐rank testing (Figure 5G) revealed significantly different patient prognoses associated with each cluster.

Specifically, C1 correlated with poorer outcomes, underscoring the clinical relevance of this immune‐guided taxonomy. Consensus map‐based assignment classified patients (Figure 5D, Supporting Information S7).

Although our immune‐informed classification was developed independently of existing frameworks, we conceptually compared the two clusters (C1/C2) with the established TCGA molecular subtypes (EBV‐positive, MSI‐high, genomically stable, and chromosomal instability). The C1 subgroup appeared more consistent with genomically stable and chromosomal instability patterns, typically associated with less immune infiltration, whereas C2 shared features of MSI‐high and EBV‐positive tumors, which generally exhibit stronger immune activation. These distinctions suggest that our classification captures immunological dimensions complementary to traditional molecular subtyping, thereby offering an additional layer of biological and clinical insight.

In summary, by integrating immunogenetic, transcriptomic and clinical data, this study constructed an immune‐informed molecular classification of gastric cancer holding promise to predict outcomes and identify immunotherapy targets. Further studies are needed to translate these findings into precision oncology applications.

### 3.9 Characterizing the Tumor Immune Microenvironment of Gastric Cancer Molecular Subtypes

**FIGURE 6:**
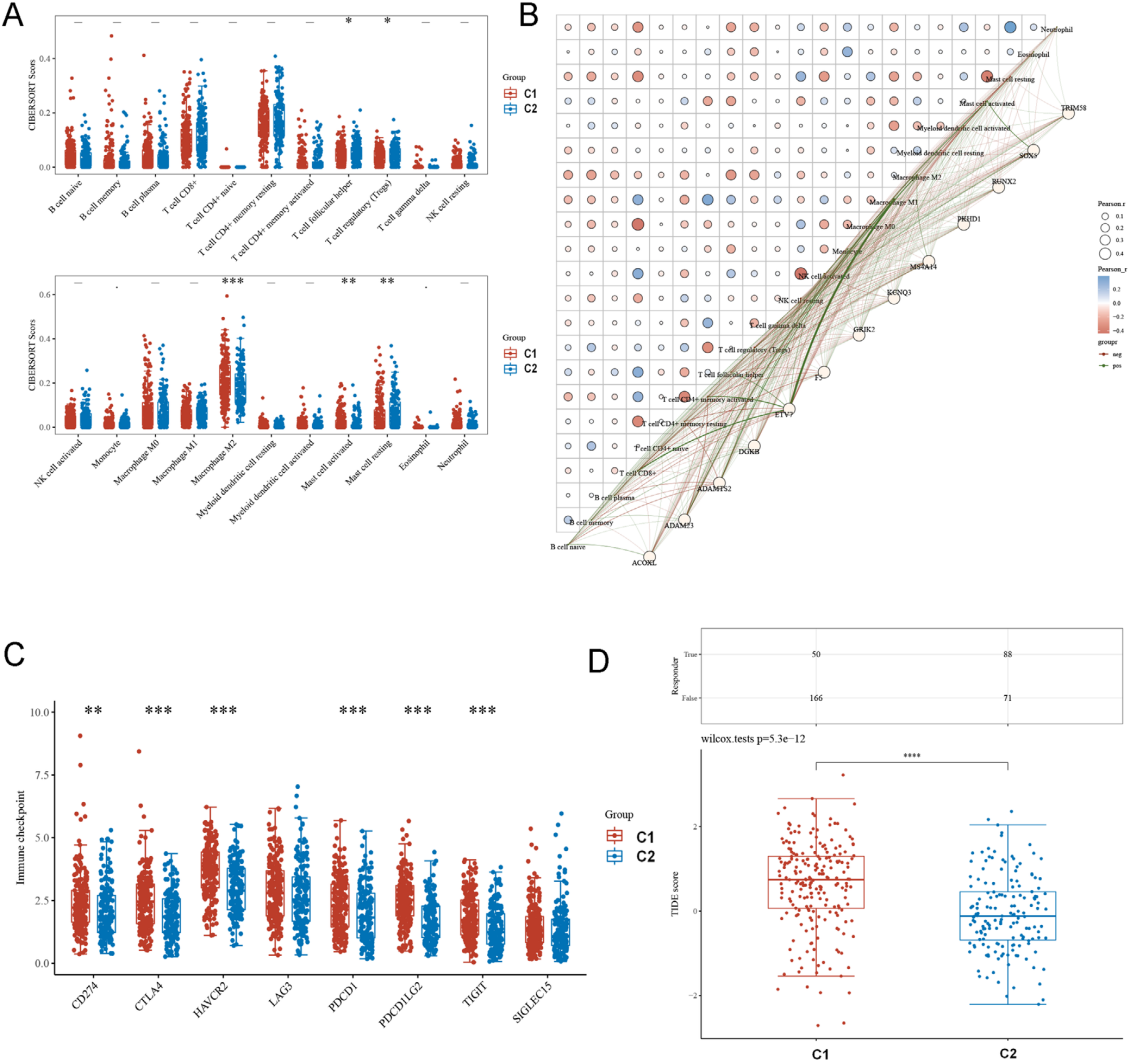
Characterization of the tumor immune microenvironment for gastric cancer molecular subtypes. (A) Subtype classification associated significantly with immune cell populations, with macrophages in the C1 cluster exhibiting elevated immune scores versus C2. (B) Correlation network analysis probed relationships between immune phenotype gene expression and scores. (C) Poor prognosis C1 patients demonstrated upregulated checkpoint genes, suggesting immune evasion. (D) The TIDE algorithm further predicted lower efficacy of immune checkpoint blockade therapy based on higher TIDE scores for C1 versus C2.

Subtype classification associated significantly with immune cell populations, including follicular helper T cells, regulatory T cells (Tregs), M2 macrophages and mast cells (activated and resting). Macrophages in the C1 cluster exhibited elevated immune scores versus C2 (Figure 6A).

Correlation network analysis probed relationships between immune phenotype gene expression and scores (Figure 6B). Notably, poor prognosis C1 patients demonstrated upregulated checkpoint genes, suggesting immune evasion (Figure 6C).

The TIDE algorithm further predicted lower efficacy of immune checkpoint blockade therapy based on higher TIDE scores for C1 versus C2 (Figure 6D).

Collectively, these immune profiling insights point to distinct tumor‐immune landscapes and therapeutic vulnerabilities between newly defined GC subgroups. The subtype‐immunophenotype links uncovered here warrant exploration of subgroup‐specific immunomodulatory strategies and biomarkers to improve clinical management of gastric cancer.

### 3.10 Characterizing an Immunophenotype‐Derived Prognostic Signature and the Role of ACOXL in Gastric Cancer Immune Evasion

**FIGURE 7:**
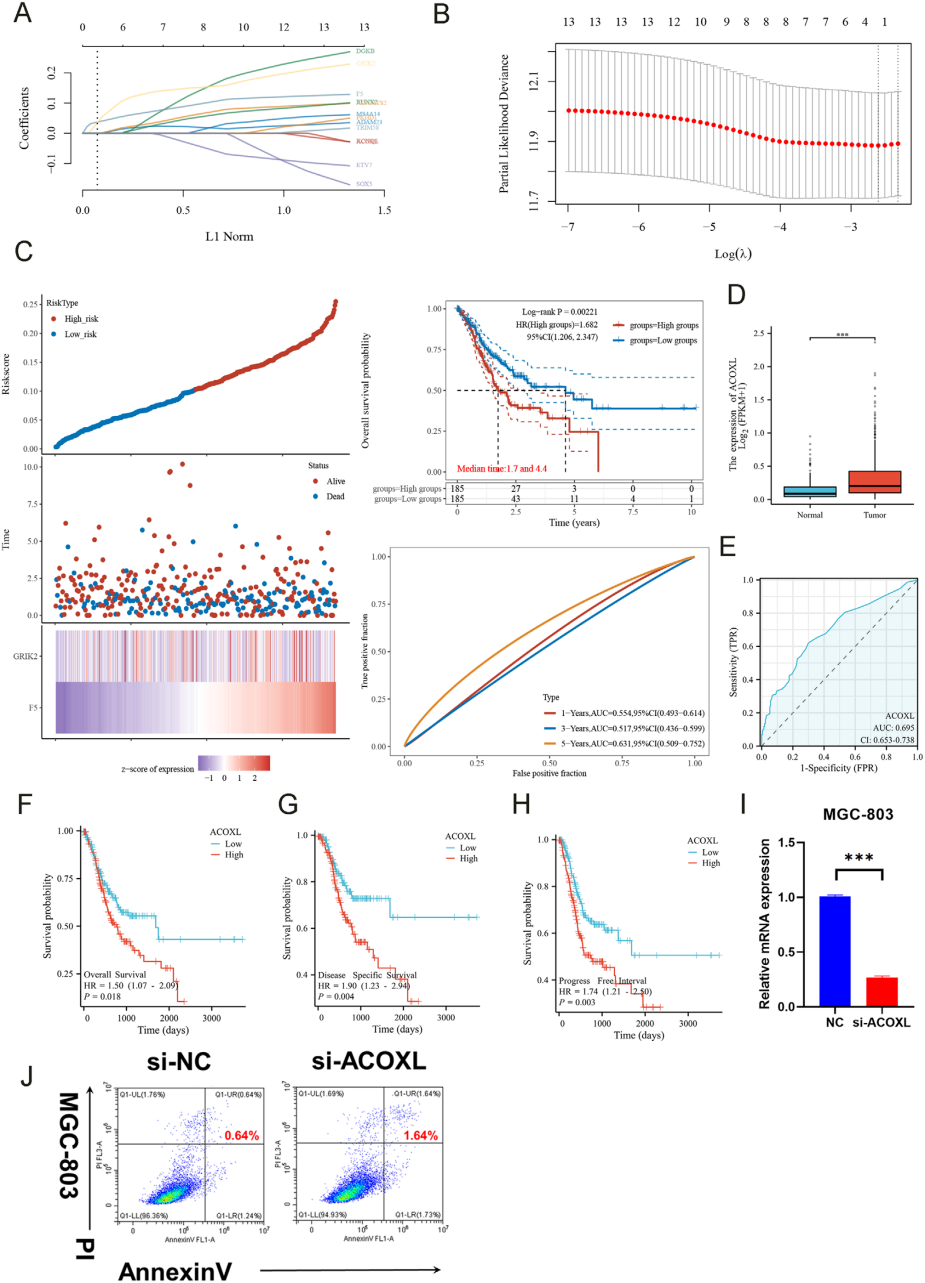
Characterization of an immunophenotype‐derived prognostic signature and the role of ACOXL in gastric cancer immune evasion. (A, B) Cox regression and LASSO modeling identified a 13‐gene signature associated with overall survival in gastric cancer patients. (C) Stratifying patients via this immunophenotype‐derived signature significantly differentiated low and high‐risk groups for mortality (HR 1.682, *p* = 0.002). (D) Particular focus on ACOXL revealed elevated tumor expression versus normal tissue. (E) Additional association with prognosis was shown using ROC. (F–H) Kaplan–Meier analyses for overall, disease‐specific and progression‐free survival. (I) In vitro, MGC‐803 cells were transfected with ACOXL siRNA, confirming knockdown. (J) Apoptosis assays demonstrated fewer apoptotic cells with ACOXL inhibition.

Cox regression and LASSO modeling identified a 13‐gene signature associated with overall survival in gastric cancer patients (Figure 7A,B). The prognostic risk score equation was: Riskscore = (0.0324) × F5 + (0.0088) × GRIK2.

Stratifying patients via this immunophenotype‐derived signature significantly differentiated low and high‐risk groups for mortality (HR 1.682, *p* = 0.002). Receiver operating curves validated accuracy up to 5 years (Figure 7C).

Particular focus on ACOXL revealed elevated tumor expression versus normal tissue (Figure 7D). Additional association with prognosis was shown using ROC (AUC 0.695, Figure 7E) and Kaplan–Meier analyses for overall, disease‐specific and progression‐free survival (Figure 7F–H).

In vitro, MGC‐803 cells were transfected with ACOXL siRNA, confirming knockdown (Figure 7I). Apoptosis assays then demonstrated fewer apoptotic cells respectively with ACOXL inhibition (Figure 7J).

Collectively, these analyses characterize immune‐informed survival signatures within GC subgroups. Findings implicate ACOXL in gastric tumorigenesis, warranting further elucidation of mechanisms driving immune evasion and prognosis.

## 4 Discussion

In this study, we employed a diversity of analytical approaches to characterize immunological attributes related to gastric cancer susceptibility and prognosis. Leveraging large genomic datasets, Mendelian randomization analyses identified potential causal relationships between alcohol intake, immune cell profiles and gastric cancer risk. Moreover, multivariable Mendelian randomization provided evidence that higher natural killer cell percentages may lower cancer risk, while alcohol intake slightly increases risk, with naive‐mature B cells potentially mediating alcohol's effect.

Alcohol may promote cancer development through mechanisms such as activation of inflammatory responses and cellular signaling pathways (Bala et al. 2016; Cho et al. 2018; Gao et al. 2019). NK cells are immune cells capable of directly killing tumor cells (Cooley et al. 2018; Ramakrishnan et al. 2019). In our study, a marginally significant negative relationship was observed between alcohol intake and NK cell frequency (OR = 0.9377, *p* = 0.0359). While the result is not strongly significant, it aligns with existing literature suggesting that alcohol may modulate immune responses by impairing NK cell function (Ruiz‐Cortes et al. 2022). NK cells are pivotal in immune surveillance, and alcohol‐induced alterations in NK cell activity could contribute to a reduced ability to target and eliminate cancerous cells. This phenomenon is supported by previous studies indicating that chronic alcohol consumption leads to decreased NK cell cytotoxicity and number, potentially compromising the immune system's capacity to respond to malignancies (Im et al. 2016; Kim et al. 2022). While our findings are exploratory, they provide important preliminary evidence that alcohol intake may indirectly influence cancer risk by modulating immune cell populations, such as NK cells. Further studies with larger sample sizes and refined methodologies are needed to confirm these results and elucidate the underlying mechanisms.

In this hypothesis, naive‐mature B cells are suggested to play a role in mediating the effects of alcohol on cancer risk. These cells are part of the immune system and contribute to antibody production and immune responses (Deenick et al. 2013; Galvani et al. 2022; Wang et al. 2020). Alcohol intake may disrupt the normal function of naive‐mature B cells, affecting the immune system's ability to monitor and control cancer cells. It is important to note that further research is needed to validate and better understand the mechanisms involved in this hypothesis. The immune system and cancer development are complex processes influenced by multiple factors. Therefore, maintaining a healthy lifestyle, including moderate alcohol consumption, a balanced diet, and regular check‐ups, is crucial in reducing the risk of cancer.

Subsequent SNP annotation elucidated 104 host genes linked to immunophenotype loci. These findings begin to uncover molecular mechanisms connecting immune regulation to tumourigenesis. Unbiased transcriptomic classification revealed two gastric cancer subgroups with distinct immune landscapes and clinical outcomes. Subtype‐immunophenotype links warrant optimizing immunotherapies. These findings could significantly influence clinical decision‐making, particularly in the context of personalized medicine. For example, immune signatures associated with high cancer risk could be modulated by immunotherapies targeting specific immune cell populations or metabolic pathways, potentially reversing immune suppression caused by alcohol. By enhancing NK cell activity or improving the function of naive‐mature B cells, clinicians could develop tailored interventions to restore effective anti‐tumor immunity, thereby improving patient outcomes. In addition, modulating the immune signatures we identified could allow for more precise monitoring of treatment response, particularly for patients at high risk due to alcohol consumption. These approaches may guide the development of new therapeutic strategies aimed at mitigating the adverse effects of alcohol on immune function and preventing cancer progression.

Combining immunogenomic and clinical data, we derived a prognostic signature stratifying patient survival based on immune functional reprogramming. Focusing on ACOXL, elevated tumor expression was associated with poorer prognosis. ACOXL encodes a peroxisomal acyl‐CoA oxidase‐like protein implicated in very‐long‐chain fatty‐acid β‐oxidation and broader lipid metabolic homeostasis (Li et al. 2022); through effects on cellular redox balance and membrane‐lipid remodeling, ACOXL has been linked to the regulation of apoptosis (He et al. 2021). In vitro experiments implicated ACOXL in cancer cell evasion of apoptosis, supporting a role in pathogenesis. Taken together, these observations suggest that ACOXL‐driven perturbations of lipid catabolism may couple metabolic reprogramming to apoptosis resistance and immune evasion within the gastric tumor microenvironment, providing a mechanistic context for its adverse prognostic association. This insight into the role of ACOXL in immune evasion and tumor metabolism could guide the development of novel therapeutic strategies. By targeting metabolic pathways involving ACOXL, it may be possible to reverse the immune evasion and apoptosis resistance seen in gastric cancer, providing new avenues for treatment that directly address the metabolic reprogramming associated with cancer progression.

However, several limitations exist. Causal inferences from Mendelian randomization rely on instrument validity assumptions, and larger datasets are needed to corroborate immune cell and alcohol relationships. Mechanisms linking candidate genes to tumor immunity also require experimental validation. One notable limitation of our study is that the data analyzed were primarily derived from populations of European ancestry. This limits the generalizability of our findings to other ethnic groups, as genetic and environmental factors influencing gastric cancer and immune response may differ across populations. Further research incorporating diverse cohorts from various ethnic backgrounds is essential to assess whether the identified immune signatures and genetic associations hold true across different populations.

Additionally, our study did not apply multiple testing corrections for the large number of immune phenotypes tested, which could increase the risk of false positives or negatives. It is also important to consider that the statistical power of some of our analyses, particularly for the marginally significant results, may have been insufficient. This limitation could be due to factors such as small sample sizes for certain immune phenotypes, potential measurement error in immune cell profiles, or the inherent complexity of the relationships between alcohol consumption, immune cells, and gastric cancer risk. The exploratory nature of our study, coupled with the wide range of immune phenotypes assessed, means that some findings may have been underpowered to detect true associations. Larger studies with more robust sample sizes and improved methodologies, such as replication cohorts or more refined immune profiling, are essential to validate these findings and provide clearer insights into the biological mechanisms underlying these relationships.

In conclusion, by integrating multi‐omics profiling with epidemiology, our study provides genetic and functional evidence that immunity and environmental factors impact gastric cancer. These findings contribute to a deeper understanding of the disease's etiology and highlight the importance of immune and metabolic signatures in shaping cancer risk and progression. This integration advances comprehension of disease etiology, offering potential avenues for improving prevention and precision medicine strategies. In clinical practice, these findings could inform alcohol counseling for high‐risk patients. For example, patients identified with immune signatures that are strongly influenced by alcohol consumption could benefit from personalized counseling and interventions aimed at reducing alcohol intake. Such approaches would help mitigate the adverse effects of alcohol on the immune system and potentially reduce the risk of gastric cancer. However, further research with larger datasets and more diverse populations is necessary to confirm the findings and clarify the underlying biological mechanisms. Ultimately, the incorporation of immune and metabolic biomarkers into clinical oncology will facilitate more effective and personalized treatment regimens, potentially improving patient outcomes and optimizing therapeutic interventions. Overall, this multidisciplinary investigation enhances the recognition of modifiable disease determinants across biological scales, paving the way for more targeted and individualized clinical care.

## Author Contributions

**Ke‐Jie He:** conceptualization, methodology, software, data curation, supervision, formal analysis, validation, investigation, funding acquisition, visualization, project administration, resources, writing – original draft, writing – review and editing. **Haitao Wang:** conceptualization, methodology.

## Funding

This project was funded by 82505254/National Nature Science Foundation of China; 2025KY1779/Technology Project of Zhejiang Provincial Health Commission; 2023K117/Quzhou Municipal Science and Technology Bureau.

## Ethics Statement

The authors have nothing to report.

## Consent

The authors have nothing to report.

## Conflicts of Interest

The authors declare no conflicts of interest.

## Supporting information


**Supporting Information: S1.** Details of Mendelian randomization analysis assessing the causal effect of alcohol consumption on gastric cancer risk.

**Supporting Information: S2.** Full list of immune cell profiles significantly associated with altered gastric cancer risk.

**Supporting Information: S3.** Additional Mendelian randomization results examining whether gastric cancer causally influences immune cell phenotypes.

**Supporting Information: S4.** Extended Mendelian randomization results investigating the impact of alcohol consumption on immune cell subsets.

**Supporting Information: S5.** Detailed annotation of SNPs linked to immune traits and gastric cancer risk.

**Supporting Information: S6.** Unsupervised consensus clustering of TCGA gastric cancer transcriptomic data stratified patients into two distinct subgroups (C1 and C2) based on immune‐related gene expression patterns.

**Supporting Information: S7.** Extended survival analysis comparing patient outcomes between immune‐defined molecular subtypes.

**Table S1:** Effect of alcohol, NK cells and B cells on gastric cancer risk by multivariable Mendelian randomization.

**Table S2:** Proportion of alcohol's effect on gastric cancer risk mediated by immune cells.

## Data Availability

No new datasets were generated in this study. However, publicly available datasets were analyzed. The gastric cancer transcriptomic and clinical data were obtained from The Cancer Genome Atlas (TCGA), and summary statistics for immune traits and gastric cancer genome‐wide association studies (GWAS) were retrieved from previously published sources as cited in the manuscript. All data used in this study are publicly accessible, and detailed accession information is provided in the corresponding references.

## References

[fsn371260-bib-0001] Al‐Batran, S. E. , N. Homann , C. Pauligk , et al. 2017. “Effect of Neoadjuvant Chemotherapy Followed by Surgical Resection on Survival in Patients With Limited Metastatic Gastric or Gastroesophageal Junction Cancer: The AIO‐FLOT3 Trial.” JAMA Oncology 3, no. 9: 1237–1244. 10.1001/jamaoncol.2017.0515. 28448662 PMC5824287

[fsn371260-bib-0002] Bala, S. , T. Csak , B. Saha , et al. 2016. “The Pro‐Inflammatory Effects of miR‐155 Promote Liver Fibrosis and Alcohol‐Induced Steatohepatitis.” Journal of Hepatology 64, no. 6: 1378–1387. 10.1016/j.jhep.2016.01.035. 26867493 PMC4874886

[fsn371260-bib-0003] Bowden, J. , G. Davey Smith , P. C. Haycock , and S. Burgess . 2016. “Consistent Estimation in Mendelian Randomization With Some Invalid Instruments Using a Weighted Median Estimator.” Genetic Epidemiology 40, no. 4: 304–314. 10.1002/gepi.21965. 27061298 PMC4849733

[fsn371260-bib-0004] Burgess, S. , D. S. Small , and S. G. Thompson . 2017. “A Review of Instrumental Variable Estimators for Mendelian Randomization.” Statistical Methods in Medical Research 26, no. 5: 2333–2355. 10.1177/0962280215597579. 26282889 PMC5642006

[fsn371260-bib-0005] Califano, A. , and M. J. Alvarez . 2017. “The Recurrent Architecture of Tumour Initiation, Progression and Drug Sensitivity.” Nature Reviews. Cancer 17, no. 2: 116–130. 10.1038/nrc.2016.124. 27977008 PMC5541669

[fsn371260-bib-0006] Cho, Y. E. , L. R. Yu , M. A. Abdelmegeed , S. H. Yoo , and B. J. Song . 2018. “Apoptosis of Enterocytes and Nitration of Junctional Complex Proteins Promote Alcohol‐Induced Gut Leakiness and Liver Injury.” Journal of Hepatology 69, no. 1: 142–153. 10.1016/j.jhep.2018.02.005. 29458168 PMC6008177

[fsn371260-bib-0007] Chyou, P. H. , A. M. Nomura , J. H. Hankin , and G. N. Stemmermann . 1990. “A Case‐Cohort Study of Diet and Stomach Cancer.” Cancer Research 50, no. 23: 7501–7504. 2253198

[fsn371260-bib-0008] Cooley, S. , P. Parham , and J. S. Miller . 2018. “Strategies to Activate NK Cells to Prevent Relapse and Induce Remission Following Hematopoietic Stem Cell Transplantation.” Blood 131, no. 10: 1053–1062. 10.1182/blood-2017-08-752170. 29358179 PMC5863700

[fsn371260-bib-0009] Deenick, E. K. , D. T. Avery , A. Chan , et al. 2013. “Naive and Memory Human B Cells Have Distinct Requirements for STAT3 Activation to Differentiate Into Antibody‐Secreting Plasma Cells.” Journal of Experimental Medicine 210, no. 12: 2739–2753. 10.1084/jem.20130323. 24218138 PMC3832925

[fsn371260-bib-0010] Galvani, R. G. , S. M. Perobelli , T. Gonçalves‐Silva , et al. 2022. “Mature Naive B Cells Regulate the Outcome of Murine Acute Graft‐Versus‐Host Disease in an IL‐10‐Independent Manner.” Transplant Cell Therapy 28, no. 4: 181.e181–e189. 10.1016/j.jtct.2022.01.004. 35032717

[fsn371260-bib-0011] Gao, B. , M. F. Ahmad , L. E. Nagy , and H. Tsukamoto . 2019. “Inflammatory Pathways in Alcoholic Steatohepatitis.” Journal of Hepatology 70, no. 2: 249–259. 10.1016/j.jhep.2018.10.023. 30658726 PMC6361545

[fsn371260-bib-0012] Hanahan, D. , and R. A. Weinberg . 2011. “Hallmarks of Cancer: The Next Generation.” Cell 144, no. 5: 646–674. 10.1016/j.cell.2011.02.013. 21376230

[fsn371260-bib-0013] Hartwig, F. P. , G. Davey Smith , and J. Bowden . 2017. “Robust Inference in Summary Data Mendelian Randomization via the Zero Modal Pleiotropy Assumption.” International Journal of Epidemiology 46, no. 6: 1985–1998. 10.1093/ije/dyx102. 29040600 PMC5837715

[fsn371260-bib-0014] He, S. , F. Lyu , L. Lou , et al. 2021. “Anti‐Tumor Activities of *Panax quinquefolius* Saponins and Potential Biomarkers in Prostate Cancer.” Journal of Ginseng Research 45, no. 2: 273–286. 10.1016/j.jgr.2019.12.007. 33841008 PMC8020356

[fsn371260-bib-0015] Heo, M. J. , T. H. Kim , J. S. You , D. Blaya , P. Sancho‐Bru , and S. G. Kim . 2019. “Alcohol Dysregulates miR‐148a in Hepatocytes Through FoxO1, Facilitating Pyroptosis via TXNIP Overexpression.” Gut 68, no. 4: 708–720. 10.1136/gutjnl-2017-315123. 29475852 PMC6581021

[fsn371260-bib-0016] Im, H. J. , H. G. Kim , J. S. Lee , et al. 2016. “A Preclinical Model of Chronic Alcohol Consumption Reveals Increased Metastatic Seeding of Colon Cancer Cells in the Liver.” Cancer Research 76, no. 7: 1698–1704. 10.1158/0008-5472.Can-15-2114. 26857263

[fsn371260-bib-0017] Kim, A. , C. K. Cajigas‐Du Ross , J. Dasarathy , et al. 2022. “Diminished Function of Cytotoxic T‐ and NK‐ Cells in Severe Alcohol‐Associated Hepatitis.” Metabolism and Target Organ Damage 2, no. 4: 18. 10.20517/mtod.2022.13. 39148503 PMC11326509

[fsn371260-bib-0018] Ko, K. P. , S. K. Park , J. J. Yang , et al. 2013. “Intake of Soy Products and Other Foods and Gastric Cancer Risk: A Prospective Study.” Journal of Epidemiology 23, no. 5: 337–343. 10.2188/jea.je20120232. 23812102 PMC3775527

[fsn371260-bib-0019] Latif, O. , J. D. Peterson , and C. Waltenbaugh . 2002. “Alcohol‐Mediated Polarization of Type 1 and Type 2 Immune Responses.” Frontiers in Bioscience 7: a135–a147. 10.2741/latif. 12133821

[fsn371260-bib-0020] Li, M. , Y. Wang , Z. Tang , et al. 2022. “Expression Plasticity of Peroxisomal Acyl‐Coenzyme A Oxidase Genes Implies Their Involvement in Redox Regulation in Scallops Exposed to PST‐Producing Alexandrium.” Marine Drugs 20, no. 8: 472. 10.3390/md20080472. 35892940 PMC9332717

[fsn371260-bib-0021] Lundberg, J. C. , and S. D. Passik . 1997. “Alcohol and Cancer: A Review for Psycho‐Oncologists.” Psychooncology 6, no. 4: 253–266. 10.1002/(sici)1099-1611(199712)6:4<253::Aid-pon276>3.0.Co;2-3. 9451745

[fsn371260-bib-0022] Mathews, S. , D. Feng , I. Maricic , C. Ju , V. Kumar , and B. Gao . 2016. “Invariant Natural Killer T Cells Contribute to Chronic‐Plus‐Binge Ethanol‐Mediated Liver Injury by Promoting Hepatic Neutrophil Infiltration.” Cellular & Molecular Immunology 13, no. 2: 206–216. 10.1038/cmi.2015.06. 25661730 PMC4786627

[fsn371260-bib-0023] Meadows, G. G. , M. Wallendal , A. Kosugi , J. Wunderlich , and D. S. Singer . 1992. “Ethanol Induces Marked Changes in Lymphocyte Populations and Natural Killer Cell Activity in Mice.” Alcoholism, Clinical and Experimental Research 16, no. 3: 474–479. 10.1111/j.1530-0277.1992.tb01403.x. 1626648

[fsn371260-bib-0024] Nagy, L. E. , W. X. Ding , G. Cresci , P. Saikia , and V. H. Shah . 2016. “Linking Pathogenic Mechanisms of Alcoholic Liver Disease With Clinical Phenotypes.” Gastroenterology 150, no. 8: 1756–1768. 10.1053/j.gastro.2016.02.035. 26919968 PMC4887335

[fsn371260-bib-0025] Orrù, V. , M. Steri , C. Sidore , et al. 2020. “Complex Genetic Signatures in Immune Cells Underlie Autoimmunity and Inform Therapy.” Nature Genetics 52, no. 10: 1036–1045. 10.1038/s41588-020-0684-4. 32929287 PMC8517961

[fsn371260-bib-0026] Rahman, R. , A. W. Asombang , and J. A. Ibdah . 2014. “Characteristics of Gastric Cancer in Asia.” World Journal of Gastroenterology 20, no. 16: 4483–4490. 10.3748/wjg.v20.i16.4483. 24782601 PMC4000485

[fsn371260-bib-0027] Ramakrishnan, S. , V. Granger , M. Rak , et al. 2019. “Inhibition of EZH2 Induces NK Cell‐Mediated Differentiation and Death in Muscle‐Invasive Bladder Cancer.” Cell Death and Differentiation 26, no. 10: 2100–2114. 10.1038/s41418-019-0278-9. 30692641 PMC6748105

[fsn371260-bib-0028] Ruiz‐Cortes, K. , D. N. Villageliu , and D. R. Samuelson . 2022. “Innate Lymphocytes: Role in Alcohol‐Induced Immune Dysfunction.” Frontiers in Immunology 13: 934617. 10.3389/fimmu.2022.934617. 36105802 PMC9464604

[fsn371260-bib-0029] Saha, B. , J. C. Bruneau , K. Kodys , and G. Szabo . 2015. “Alcohol‐Induced miR‐27a Regulates Differentiation and M2 Macrophage Polarization of Normal Human Monocytes.” Journal of Immunology 194, no. 7: 3079–3087. 10.4049/jimmunol.1402190. PMC4517579 25716995

[fsn371260-bib-0030] Sakaue, S. , M. Kanai , Y. Tanigawa , et al. 2021. “A Cross‐Population Atlas of Genetic Associations for 220 Human Phenotypes.” Nature Genetics 53, no. 10: 1415–1424. 10.1038/s41588-021-00931-x. 34594039 PMC12208603

[fsn371260-bib-0031] Sanderson, E. 2021. “Multivariable Mendelian Randomization and Mediation.” Cold Spring Harbor Perspectives in Medicine 11, no. 2: a038984. 10.1101/cshperspect.a038984. 32341063 PMC7849347

[fsn371260-bib-0032] Schreiber, R. D. , L. J. Old , and M. J. Smyth . 2011. “Cancer Immunoediting: Integrating Immunity's Roles in Cancer Suppression and Promotion.” Science 331, no. 6024: 1565–1570. 10.1126/science.1203486. 21436444

[fsn371260-bib-0033] Sidore, C. , F. Busonero , A. Maschio , et al. 2015. “Genome Sequencing Elucidates Sardinian Genetic Architecture and Augments Association Analyses for Lipid and Blood Inflammatory Markers.” Nature Genetics 47, no. 11: 1272–1281. 10.1038/ng.3368. 26366554 PMC4627508

[fsn371260-bib-0034] Spitzer, J. H. , and G. G. Meadows . 1999. “Modulation of Perforin, Granzyme A, and Granzyme B in Murine Natural Killer (NK), IL2 Stimulated NK, and Lymphokine‐Activated Killer Cells by Alcohol Consumption.” Cellular Immunology 194, no. 2: 205–212. 10.1006/cimm.1999.1511. 10383823

[fsn371260-bib-0035] Thompson, E. D. , M. Zahurak , A. Murphy , et al. 2017. “Patterns of PD‐L1 Expression and CD8 T Cell Infiltration in Gastric Adenocarcinomas and Associated Immune Stroma.” Gut 66, no. 5: 794–801. 10.1136/gutjnl-2015-310839. 26801886 PMC4958028

[fsn371260-bib-0036] Tramacere, I. , E. Negri , C. Pelucchi , et al. 2012. “A Meta‐Analysis on Alcohol Drinking and Gastric Cancer Risk.” Annals of Oncology 23, no. 1: 28–36. 10.1093/annonc/mdr135. 21536659

[fsn371260-bib-0037] Van Cutsem, E. , X. Sagaert , B. Topal , K. Haustermans , and H. Prenen . 2016. “Gastric Cancer.” Lancet 388, no. 10060: 2654–2664. 10.1016/s0140-6736(16)30354-3. 27156933

[fsn371260-bib-0038] Verma, V. K. , H. Li , R. Wang , et al. 2016. “Alcohol Stimulates Macrophage Activation Through Caspase‐Dependent Hepatocyte Derived Release of CD40L Containing Extracellular Vesicles.” Journal of Hepatology 64, no. 3: 651–660. 10.1016/j.jhep.2015.11.020. 26632633 PMC4761285

[fsn371260-bib-0039] Wang, H. , H. C. Morse 3rd , and S. Bolland . 2020. “Transcriptional Control of Mature B Cell Fates.” Trends in Immunology 41, no. 7: 601–613. 10.1016/j.it.2020.04.011. 32446878

[fsn371260-bib-0040] Yavorska, O. O. , and S. Burgess . 2017. “Mendelian Randomization: An R Package for Performing Mendelian Randomization Analyses Using Summarized Data.” International Journal of Epidemiology 46, no. 6: 1734–1739. 10.1093/ije/dyx034. 28398548 PMC5510723

[fsn371260-bib-0041] Zheng, J. , D. Baird , M. C. Borges , et al. 2017. “Recent Developments in Mendelian Randomization Studies.” Current Epidemiology Reports 4, no. 4: 330–345. 10.1007/s40471-017-0128-6. 29226067 PMC5711966

[fsn371260-bib-0042] Zhu, H. , X. Yang , C. Zhang , et al. 2013. “Red and Processed Meat Intake Is Associated With Higher Gastric Cancer Risk: A Meta‐Analysis of Epidemiological Observational Studies.” PLoS One 8, no. 8: e70955. 10.1371/journal.pone.0070955. 23967140 PMC3743884

