# Supplementary material for: Unraveling the Interplay Between Alcohol, Immunity, and Gastric Cancer: A Genomic Approach
Source: Food Sci Nutr. 2025 Nov 24;13(12):e71260. doi: 10.1002/fsn3.71260 (PMC12641442; doi:10.1002/fsn3.71260)
Supplement: Supplementary file 8 — Table S1: Effect of alcohol, NK cells and B cells on gastric cancer risk by multivariable Mendelian randomization. [file FSN3-13-e71260-s008.docx]

|  |  |  |  |  |  |
| --- | --- | --- | --- | --- | --- |
| Exposure | Outcome | or | or_lci95 | or_uci95 | pval |
| Naive-mature B cell %B cell | Gastric cancer | 1.002 | 0.955 | 1.05 | 0.91 |
| Natural Killer %lymphocyte | Gastric cancer | 0.962 | 0.933 | 0.99 | 0.01 |
| Alcohol consumption | Gastric cancer | 1.046 | 1.001 | 1.09 | 0.04 |

**Table 1: Effect of alcohol consumption and immune cells on gastric cancer by multivariable mendelian randomization（MVMR）**
