# Supplementary material for: Unraveling the Interplay Between Alcohol, Immunity, and Gastric Cancer: A Genomic Approach
Source: Food Sci Nutr. 2025 Nov 24;13(12):e71260. doi: 10.1002/fsn3.71260 (PMC12641442; doi:10.1002/fsn3.71260)
Supplement: Supplementary file 9 — Table S2: Proportion of alcohol's effect on gastric cancer risk mediated by immune cells. [file FSN3-13-e71260-s003.docx]

|  | PM | PM_lci95 | PM_uci95 | pval |
| --- | --- | --- | --- | --- |
| Naive-mature B cell %B cell | 0.109708 | 0.035823 | 0.183593 | 0.035346 |
| Natural Killer %lymphocyte | 0.051016 | 0.008235 | 0.093797 | 0.065856 |
|  |  |  |  |  |

**Table 2: Estimate of the effect of alcohol consumption on gastric cancer explained by naive-mature B cells and NK cells**

PM: proportion mediated
